# Investigating geographical variation in the use of mental health services by area of England: a cross-sectional ecological study

**DOI:** 10.1186/s12913-021-06976-2

**Published:** 2021-09-11

**Authors:** Lucy Maconick, Luke Sheridan Rains, Rebecca Jones, Brynmor Lloyd-Evans, Sonia Johnson

**Affiliations:** Division of Psychiatry, University College London, 6th Floor Maple House, 149 Tottenham Court Road, London, W1T 7NF United Kingdom

**Keywords:** Mental health, Mental health services, Geographical variation, Access to services

## Abstract

**Background:**

There is evidence of geographical variation in the use of mental health services in the UK and in international settings. It is important to understand whether this variation reflects differences in the prevalence of mental disorders, or if there is evidence of variation related to other factors, such as population socioeconomic status and access to primary care services.

**Methods:**

This is a cross-sectional ecological study using Public Health England data. The unit of analysis was the population served by clinical commissioning groups (CCGs), National Health Service (NHS) catchment areas. The analysis explored associations between area characteristics and the number of people in contact with mental health services using regression modelling. Explanatory variables included age, gender, prevalence of severe mental illness (SMI), prevalence of common mental disorder (CMD), index of multiple deprivation (IMD), unemployment, proportion of the population who are Black and Minority Ethnic (BAME), population density, access to and recovery in primary care psychological therapies. Unadjusted results are reported, as well as estimates adjusted for age, prevalence of CMD and prevalence of SMI.

**Results:**

The populations of 194 CCGs were included, clustered within 62 trusts (NHS providers of mental health services). The number of people in contact with mental health services showed wide variation by area (range from 1131 to 5205 per 100,000 population). Unemployment (adjusted IRR 1.11; 95% CI 1.05 to 1.17; *p* < 0.001) and deprivation (adjusted IRR 1.02 95% CI 1.01 to 1.04; p < 0.001) were associated with more people being in contact with mental health services. Areas with a higher proportion of the population who are BAME (IRR 0.95 95% CI 0.92 to 0.99 *p* = 0.007) had lower service use per 100,000 population. There was no evidence for association with access to primary care psychological therapies.

**Conclusions:**

There is substantial variation in the use of mental health services by area of England. Social factors including deprivation, unemployment and population ethnicity continued to be associated with the outcome after controlling for the prevalence of mental illness. This suggests that there are factors that influence the local population use of mental health services in addition to the prevalence of mental disorder.

**Supplementary Information:**

The online version contains supplementary material available at 10.1186/s12913-021-06976-2.

## Background

Geographical variation in the use of mental health services has been observed in multiple international settings [1–4] [5]. It is important to understand whether this variation reflects differences in the prevalence of mental disorders, if it is related to population characteristics such as socioeconomic status and ethnicity, or whether access to services rather than need might be associated with this variation. There is little previous research in this area in Europe. In the US Medicaid system, interstate variations in mental health spending (a proxy for health service use) have been found to exceed the interstate variations in Medicaid spending for inpatient, outpatient, pharmacy and acute services for other disorders [2]. In this work the authors acknowledge that in the extensive literature on the geographical variation in use of health services, mental health services have been relatively neglected.

Public data shows substantial variation in the number of people in contact with specialist mental health services in England [6]. We have not found published investigations of the factors associated with these variations, although some studies have focused on factors that might explain geographical differences in the prevalence of mental disorders [7] [8] [9] . Factors found to be associated with higher prevalence of mental disorders have included higher population density [10], [11], unemployment [12], urban location [13] [14] and a higher number of single person households [8]. The incidence of schizophrenia in non-white ethnic minorities has found to be greater in areas where ethnic minorities make up a smaller proportion of the population [15]. There is also an extensive literature looking at how an individual’s local environment may influence individual risk of both psychosis and common mental disorders [8] [16], with mixed results.

Service use is likely to be influenced not only by the prevalence of mental disorder, but also by factors that affect health service utilisation, such as service access and health help-seeking behaviour. The Care Quality Commission (CQC) has commented on the unacceptable variation in mental health service provision across England [17]. Area level factors identified that may influence the proportion of people with a mental disorder who receive treatment include population density [18], urban-rural location [19] and ethnicity [20] [21]. A study that compared estimated prevalence rates of mental disorder with observed treatment rates in primary care in small areas of England found that ethnicity was a consistent predictor of geographical variations in service use relative to need [22], with areas with higher proportion of black populations having significantly lower numbers of people in treatment for depression than the expected prevalence, suggesting reduced help seeking behaviour or poorer access to services for this group. The authors also observed a pronounced ‘London effect’, where the number of people being treated by primary care for depression in London was found to be much lower than expected on the basis of modelled estimates [22].

Additionally, different populations may show differences in health seeking behaviours. Some individual level characteristics have been associated with higher health service utilisation for common mental disorders including comorbidity, female gender, marital status of divorced, non-white ethnicity, high previous health service utilisation and lower level of functioning [23] [24]. At an individual level there is also good evidence of an association between accessing a primary care psychological treatment service and decreased health service utilisation for mental and physical disorders [23].

Understanding the influences on numbers of people in contact with specialist mental health services is of particular interest in the context of findings of a rise in the numbers in contact with mental health services over the past 10 years [25], an area of interest for policy makers and politicians [26] [6]. This rise in secondary mental health care use appears to have continued despite an increase in mental health service provision in primary care in the last decade, through increased availability of CBT and other psychological therapies through the Improving Access to Psychological Therapies program (IAPT) [27].

This study will look at whether there are area level factors associated with higher use of secondary mental health services, in addition to the local prevalence of mental disorders. This may point towards factors that result in greater demand for formal mental health services as opposed to other forms of support, which could be helpful for planning of future service provision. If there are area characteristics that are associated with lower uptake of mental health services for the same burden of mental illness, this may also point towards inequalities in access to care. Given the significant investment in primary care psychological therapies in England in the past decade, it is also interesting to consider whether areas with greater access to high quality primary care psychological therapies have a lower number of people in contact with secondary services.

### Aim and study approach

The aim of this study is to investigate the association between area level factors and the number of people in contact with secondary mental health services by catchment area (Clinical Commissioning Group) in England.

In particular we will investigate whether there are area level factors associated with the use of secondary mental health services, after controlling for the prevalence of mental disorder.

We will also investigate whether greater access to primary care psychological therapies is associated with lower use of secondary mental health services.

## Methods

The study used a cross sectional ecological study design with the resident population served by each CCG as the unit of analysis. CCGs are NHS bodies with responsibility for planning and commissioning services for a defined catchment area and cover an average population of 250,000 people [28].

In accordance with the recording of data by Public Health England (PHE), secondary mental health services are defined as National Health Service (NHS) funded adult secondary mental health services and does not include services situated in primary care such as the Improving Access to Psychological Therapies (IAPT). IAPT services provide relatively rapid access to short periods of psychological treatment for anxiety and depression throughout England.

### Primary outcome and explanatory variables

The primary outcome was the number of people in contact with adult mental health services per 100,000 population aged 18+ (end of quarter snapshot Q3 2019). This was defined as the number of people with an open adult mental health care spell in NHS funded adult specialist mental health services at the end of the reporting period. Data for the primary outcome was obtained from Public Health England ‘Fingertips’ Public Health Profiles [6], which created the variable from data from the Mental Health Services Monthly Statistics. The explanatory variables included in the analysis are set out in Table 1, with the data collection time point and data source.

**Table 1:** Variables included in the study with definitions and data collection time points

Variable	Variable definition	Data collection time point	Data source
Number of people in contact with mental health services per 100,000 population^a^	People with an open Adult Mental Health Care Spell in NHS funded adult specialist mental health services at the end of the reporting period	Q3 2019	Public Health England’s Fingertips Database
Age	Median age of the population of the Clinical Commissioning Group(CCG)	Mid-2017	Office for National Statistics (ONS)
Gender	Percentage of the population who are male	2017	ONS
Prevalence of severe mental illness (SMI)	The percentage of patients with schizophrenia, bipolar affective disorder and other psychoses as recorded on general practice disease registers.	2017/2018	Public Health England’s Fingertips Database
Estimated prevalence of common mental disorders (CMD)	The estimated proportion of the population aged 16 & over who have a common mental disorder (CMD), where CMD is defined as any type of depression or anxiety, model calculations based on Adult Psychiatric Morbidity Survey (APMS) data applied to local demography	2017	Public Health England’s Fingertips Database
Index of multiple deprivation score (IMD)	Measure of relative deprivation for small areas in England, which ranks every small area in England from 1 (most deprived) to 32,844 (least deprived) on the basis of income, employment, education skills and training, health, crime, housing and services, living environment	2015	Public Health England’s Fingertips Database
Percentage unemployed	Claimant rate: Percentage of the working age population who are claiming Jobseeker’s Allowance plus those who claim Universal Credit and are required to seek work and be available for work	2018/2019	Public Health England’s Fingertips Database
Proportion who are Black and Minority Ethnic (BAME)	Number of people on the 2011 Census who stated their ethnicity as ‘not White’ as a proportion of the total number answering the ethnicity question on the 2011 Census, per 1000 population	2011 (last census)	Public Health England’s Fingertips Database
Population density	Number of people per 10 ha	2019	Calculated using ONS Standard Area Measurements
Access to Improving Access to Psychological Therapies (IAPT)	The number of people entering IAPT services as a proportion of those estimated to have anxiety and/or depression	March 2019	Public Health England’s Fingertips Database
Recovery in IAPT	This is the number of people not at ‘caseness’ (experiencing severe enough symptoms to be considered a clinical case) at their last session, as a percentage of people who were at ‘caseness’ at their first session	August 2019	Public Health England’s Fingertips Database

^a^Primary outcome

### Data sources and study population

All CCGs in England were included in the analysis. As specified in Table 1, for the majority of indicators data was obtained from the Public Health England’s Fingertips Public Health Profiles, which is a publicly available data source available through Public Health England’s website [6].

Demographic data on age and gender were obtained from the Office of National Statistics. Data on population density of CCGs was calculated using the population of CCGs from the fingertips tool divided by the area of CCGs in hectares from the Office of National Statistics Standard Area Measurements data [29].

For all variables data was obtained as close in time as possible to the 2018/2019 time point used for measurement of the primary outcome. For some variables up to date data was not available, for example, the proportion who are BAME was last collected at the 2011 census. In these cases the most recent data available was used.

There have been changes in boundaries of CCGs over the last 5 years. The boundaries used for the study were for CCGs as they stood at the end of quarter 3 year 2018–2019. For two CCGs data for some variables was not available from PHE Fingertips based on these boundaries and so they were excluded from the analysis. The mapping of CCGs onto mental health trusts had been previously performed by our research group for another project.

The primary outcome, prevalence of CMD, prevalence of SMI and the IAPT data reflect the adult population only. The percentage of people who are from black and minority ethnic group backgrounds was only available for the total population (all ages) as it was census data. Median age of the population, IMD and population density also reflected the total population of the CCG. Differences in the median age of the population of CCGs was controlled for in the model.

### Statistical analysis

We investigated associations between eight explanatory variables and the number of people in contact with mental health services within each CCG using multilevel negative binomial regression models, to account for overdispersion in the outcome. The unit of analysis was CCG, and all models included the mid-year population of the CCG from which the count was drawn as an exposure term. All models specified a random effect of mental health trust to account for similarities between CCGs within the same trust. We fitted separate univariable models for each of the explanatory variables (prevalence of CMD, prevalence of SMI, proportion BAME, population density, IMD, percentage unemployed, access to IAPT and recovery in IAPT). Multivariable models adjusting both separately and jointly for median age, prevalence of CMD and prevalence of SMI were performed to examine which factors continue to be associated with the outcome after the prevalence of mental disorders was taken into account. Gender proportion was not included as a covariate in adjusted models because we found no evidence that it was associated with the outcome. Multilevel models were felt to more appropriately capture aspects of spatial structure compared with spatial autoregression approaches, as it was expected that there would be significant similarities in service delivery between CCGs that are within the same mental health trust. As the outcome referred to secondary mental health services that can only be accessed by people confirmed to be living within the CCG catchment area, people were also not able to access services in neighbouring CCGs, reducing the risks of ‘spillover effects’ between neighbouring CCGs not within the same trusts. To assess for evidence of remaining spatial autocorrelation not captured by multilevel models, Moran’s I test was applied to residuals of the adjusted multilevel models. This was performed using a spatial weights matrix derived from publicly available data on the digital vector boundaries of CCGs in 2019 from the Office of National Statistics [30].

## Results

194 CCGs were included in the study, clustered within 62 trusts. There was complete data for the primary outcome, age, gender, proportion BAME, percentage unemployed, prevalence of CMD and recovery in IAPT. There was a small amount of missing data for the other variables of interest (< 5% of CCGs), detailed in Additional file 1: Appendix 1.

### Variation in the use of mental health services by area

**Fig. 1:**
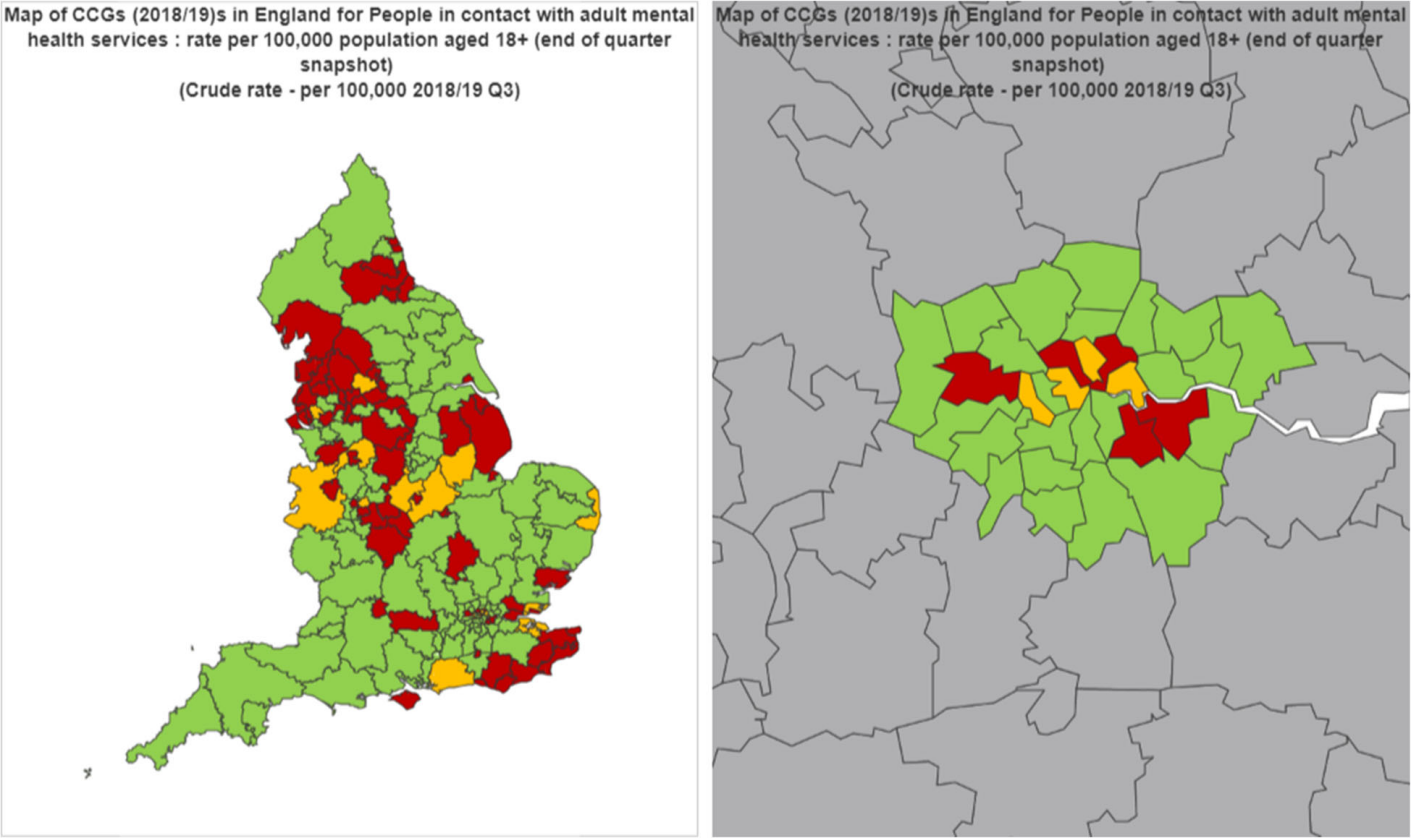
Map of the number of people in contact with mental health services by CCG in England (left) and displaying London in more detail (right) Red indicates that CCG has a higher proportion of contacts per head of population compared with the benchmark of ‘England’. Amber indicates a similar number of contacts and green indicates a lower number of contacts. Q3 = Quarter 3. Source: Fingertips Introduction 3 Public Health England. Public Health Profiles. Reproduced with permission. Accessed: 23rd October 2019 https://fingertips.phe.org.uk© Crown copyright 2020

The number of people in contact with secondary mental health services ranged from 1131 per 100,000 population in NHS Wiltshire to 5205 per 100,000 population in Greater Preston CCG. For full results see Table 2. Areas with a higher proportion of people in contact with secondary mental health services compared to the proportion for the whole of England tended to be in North West England, West Midlands and coastal areas of the South East (Fig. 1). Within London some central urban areas showed a high proportion of people in contact with mental health services compared with the rest of England.

**Table 2:** Characteristics of CCGs

	CCG Mean	SD	Range
Number of people in contact with mental health services (per 100,000)	2419	783	1131 to 5205
Age (median age of the population in years)	40.9	4.7	27.9 to 51.1
Gender (% of the population who are male)	49.4	0.8	47.6 to 52.9
Prevalence of Severe Mental Illness (SMI) (%)	0.9	0.2	0.6 to 1.5
Prevalence of Common Mental Disorders (CMD) (%)	16.9	2.7	12.0 to 24.0
Index of Multiple Deprivation Score (IMD)	22.0	8.0	7.7 to 51.5
Proportion of the population who are BAME (Black and Minority Ethnic) (people per 1000 population)	13.9	15.5	1.2 to 72.2
Unemployment rate (%)	1.9	0.2	0.5 to 4.5
Population density (people per 10 ha)	16.9	23.9	0.4 to 130.3
Access to ‘Improving Access to psychological therapy’ (IAPT) services (%)	19.4	4.4	0 to 31.2
IAPT recovery (%)	53.3	4.7	35.0 to 65.0

### Factors associated with use of mental health services

In the univariable analysis, there was strong evidence of an association between the prevalence of mental disorders and the number of people in contact with mental health services. There was also strong evidence that greater contact with mental health services is associated with a higher proportion of the population who are BAME (IRR 1.04 CI 1.01 to 1.07), greater population density (IRR 1.03 CI 1.01 to 1.05), a higher unemployment rate (IRR 1.17 CI 1.12 to 1.22) and a higher Index of Multiple Deprivation (IRR 1.02 CI 1.01 to 1.02). Table 3 displays full results of regression analyses. There was no evidence for an association between contact with mental health services and either IAPT access (IRR 1.00 CI 0.99 to 1.01) or IAPT recovery rate (IRR 1.00 CI 1.00 to 1.01).

**Table 3:** Results of regression analysis. Incidence rate ratios (IRR) are displayed with 95% confidence intervals (CI) and *p* values

Explanatory variable of interest	Unadjusted analysis *IRR (95% CI)*	Adjusted for age *IRR (95% CI)*	Adjusted for prevalence of CMD *IRR (95% CI)*	Adjusted for prevalence of SMI *IRR (95%* CI)	Adjusted for age, SMI and CMD *IRR (95%* CI)
Estimated prevalence of CMD (%)	1.05 *(1.04–1.06)*	1.05 *(1.03–1.07)*	–	1.02 *(1.01–1.04)*	1.01 *(1.10–1.04)*
	*P* < 0.001	*P* < 0.001		*P* = 0.008	*P* = 0.219*
Prevalence of SMI (%)	2.08 *(1.75–2.48)*	1.91 *(1.59–2.29)*	1.71 *(1.35–2.14)*	–	1.75 *(1.39–2.20)*
	*P* < 0.001	*P* < 0.001	*P* < 0.001		*P* < 0.001**
Proportion of population who are BAME (people per 1000 population)	1.04 *(1.01–1.07)*	0.98 *(0.94–1.02)*	0.97 *(0.94–1.01)*	1.00 (0.97–1.03)	0.95 *(0.92–0.99)*
	*P* = 0.011	*P* = 0.308	*P* = 0.114	*P* = 0.916	*P* = 0.007
Population density (number of people per 10 ha)	1.03 *(1.01–1.05)*	1.01 (0.99–1.03)	0.99 *(0.97–1.01)*	1.03 *(1.01–1.05)*	0.98 *(0.96–1.00)*
	*P* = 0.004	*P* = 0.538	*P* = 0.405	*P* = 0.003	*P* = 0.07
IMD	1.02 *(1.01–1.02)*	1.02 *(1.01–1.02)*	1.02 *(1.01–1.03)*	1.01 *(1.01–1.02)*	1.02 *(1.01–1.04)*
	*P* < 0.001	*P* < 0.001	*P* < 0.001	*P* < 0.001	*P* < 0.001
Percentage unemployed (%)	1.17 *(1.12–1.22)*	1.15 *(1.10–1.19)*	1.11 *(1.05–1.17)*	1.10 *(1.06–1.15)*	1.11 *(1.05–1.17)*
	*P* < 0.001	*P* < 0.001	*P* < 0.001	*P* < 0.001	*P* < 0.001
Access to IAPT	1.00 *(0.99–1.01)*	1.00 *(0.99–1.01)*	1.00 *(0.99–1.01)*	1.00 *(0.99–1.01)*	1.00 *(1.00–1.01)*
	*P* = 0.788	*P* = 0.995	*P* = 0.985	*P* = 0.650	*P* = 0.664
Recovery in IAPT	1.00 *(1.00–1.01)*	1.01 *(1.00–1.01)*	1.01 *(1.00–1.01)*	1.00 *(1.00–1.01)*	1.01 *(1.00–1.01)*
	*P* = 0.725	*P* = 0.130	*P* = 0.124	*P* = 0.342	*P* = 0.103

All analyses are results from negative binomial regression models. All models specified a random effect of mental health trust to account for similarities between CCGs within the same trust

*not adjusted for CMD

**not adjusted for SMI *CMD* common mental disorder, *SMI* severe mental illness, *BAME* black and minority ethnic, *IMD* index of multiple deprivation, *IAPT* Improving access to psychological therapy

After adjustment for age and the prevalence of both common and severe mental disorders, we found evidence for associations between the proportion of people in contact with mental health services and each of unemployment, deprivation, ethnicity and population density. There was strong evidence for an 11% increase in contact with mental health services for each percentage increase in unemployment (IRR 1.11 95% CI 1.05 to 1.17) and for a 2% increase in contact for each unit increase in deprivation score (1.02 95% CI 1.01 to 1.04). There was likewise strong evidence from the fully adjusted model for a 5% reduction in contact with mental health services with each additional 1 person per 1000 population who is BAME (IRR 0.95 95% CI 0.92 to 0.99), and weak evidence for a 2% reduction in contact with services for each additional 1 person per 10 ha (IRR 0.98 95% CI 0.96 to 1.00). The direction of the association with ethnicity and also with population density changed after adjusting for age. On further exploration of this, having a high proportion of the population who are BAME and a higher population density were both associated with having a younger population, suggesting that age may have confounded the relationship. There was no evidence for an association with access to IAPT or IAPT recovery rates.

Moran I’s test was applied to the residuals of adjusted multilevel models to test for evidence of remaining spatial autocorrelation not captured by the model. At a significance level of 0.05 there was no evidence found of remaining spatial autocorrelation in the residuals of the model (see Additional file 1: Appendix 2 for full results of Moran I’s test).

## Discussion

### Main findings

This study found evidence of geographical variation in the proportion of the population in contact with mental health services by area of England. There was an almost five fold difference between the area with the lowest and the area with the highest proportion of people in contact with mental health services per 100,000 population. The CCG with the largest number of people in contact with its mental health services had 2786 more patients per 100,000 population than the mean.

We found strong evidence that greater deprivation and unemployment were associated with a higher number of people in contact with mental health services, after controlling for the local prevalence of mental disorders. A higher proportion of the population who are BAME was associated with a fewer people in contact with mental health services. There was suggestive evidence that areas with a higher population density had fewer people in contact with mental health services per 100,000 population. There was no evidence for an association between access to primary care psychological therapies and the number of people in contact with secondary mental health services.

### Findings in the context of existing evidence

The observation that higher deprivation is associated with higher use of mental health services is consistent with previous studies [31]. Area deprivation may impact the number of people in contact with mental health services through affecting both the prevalence of mental disorders and health service utilization. Higher deprivation can result in higher use of formal services through increased comorbidity [32] [33], reduced social support [34] or reduced availability of non-healthcare resources to draw from [22]. There is evidence from literature that areas with higher deprivation have a higher number of compulsory admissions to mental health hospitals [35], for example, one additional percentage point in population income deprivation (according to the European Deprivation Index definition) has previously been shown to be associated with a 1.6% increase in admissions to hospital with severe mental illness [11]. It has also been highlighted in existing research that area-level deprivation is associated with mental health at the individual level, after controlling for individual level attributes, suggesting that there are factors that operate at a neighbourhood level that influence an individual’s mental health [36, 37].

Higher unemployment rates were associated with higher use of secondary mental health services. The direction of this relationship is difficult to untangle in an ecological cross-sectional study. The unemployment variable used in this analysis captured only those receiving job seekers allowance or universal credit with the requirement that the individual seeks work. It therefore did not include those who were receiving disability allowance or off work due to long term mental or physical illness, suggesting that this association cannot be explained by the area clustering of people with severe mental illness who are also long term unemployed. An ecological study does not allow us to examine whether at an individual level those who were unemployed were also those who were more likely to be in contact with mental health services, only that areas with higher levels of unemployment were also more likely to have a higher demand for mental health services. Unemployment has been previously identified as a specific risk factor for common mental disorders such as anxiety and depression [12], and so an association with primary rather than secondary care service use might be expected. There is an absence of research on the impact of unemployment on mental health service utilisation, but there is a body of research looking at the impact of unemployment on the use of general healthcare services, with evidence to suggest increased use of general health services amongst the unemployed, but also increased unmet care needs [38].

A CCG having a higher proportion of the population who are BAME was associated with a higher number of people in contact with mental health services in our univariable analysis, but the direction of this association changed when adjusting for age. Controlling for age as a confounder appeared appropriate as age was associated with the outcome and there was strong evidence that CCGs with higher median age had on average a lower proportion of the population who were BAME. The fact that areas with a higher proportion of the population who are BAME had lower service use, after the prevalence of mental disorders is taken into account, suggests that there may be differences in access to services related to ethnicity. It has been previously found that people of black African or Caribbean backgrounds are overrepresented in inpatient mental health services but underrepresented in community services [22], and that Black patients may need to navigate more complex care pathways before being accepted by secondary mental health services [39]. In contrast those from Asian backgrounds have been previously found to use inpatient units less than White patients [39]. Therefore the measure of the ethnicity of the population of CCGs would benefit from being disaggregated by different ethnicities in order to explore this relationship further.

Of interest there was no association between access and quality of primary care psychological therapy (access to IAPT and recovery in IAPT) and the number of people in contact with secondary care services. We might expect higher IAPT access may reduce referrals to secondary services, but it may be that IAPT services address the needs of a different patient group from secondary mental health services. In the NHS secondary mental health services predominantly provide care for people with schizophrenia, bipolar and complex depression with the majority of common mental disorders being cared for by GPs and IAPT services [40]. Our results contrast with those of Twomey et al., who found evidence at individual level that accessing a primary care psychological treatment service decreased health service utilisation by people with mental disorders, although this was across all aspects of healthcare [23]. Benefits of IAPT have mostly been described in terms of reduction in symptoms of mild to moderate anxiety and depression [41], reduced sick leave from work [42] and reduction in use of health services for physical health problems such as A&E [42] and so it may be that IAPT is providing valuable improvements in health for people that would not otherwise have received input from a specialist mental health service.

It was unfortunately not possible to look at the levels of mental health service coverage by area in this study. Areas with higher population coverage for mental health service are likely to have higher uptake of services relative to need [32]. De Silva describes the conditions needed for population coverage of a therapeutic intervention: it must be (i) physically available (ii) financially and geographically accessible; (iii) acceptable; (iv) used; and (v) delivered appropriately and effectively [32]. The UK aspires to fairly consistent population coverage of services, but there is regional variation in for example, access to 24 h crisis support or local availability of mental health beds [17]. In this study, there was suggestive evidence that areas with higher population density had lower numbers of people in contact with mental health services per 100,000 population once the prevalence of mental disorders was controlled for, although this was not statistically significant at the 5% level. Population density may reflect geographical proximity to services, but not necessarily population coverage, as found by Asthana and colleagues, who observed that the densely populated area of London has lower than expected uptake of services for common mental disorders [22]. There also may be service limits that constrain geographical variation in the number of people in contact with services relative to need, as in areas where demand is very high, services may not be able to expand sufficiently to meet need and so put in place higher thresholds for accepting patients. It was not possible to look at spend on mental health services in this study, which may give interesting insight into the level of prioritisation of mental health services within CCGs and possibly level of population coverage, as reported in the NHS Five Year Forward View, spending per capita across CCGs varies almost two-fold in relation to underlying need [33].

### Limitations

This is an ecological study and so conclusions cannot be drawn about individual risk factors for being in contact with mental health services. As the analysis required a multilevel negative binomial regression it was not possible to calculate the proportion of variation explained by each variable. It would have been interesting to understand the proportion of variation explained by the prevalence of mental disorder compared with the proportion explained by other factors.

It was not possible to include data on differences in the supply of mental health services, including local service coverage or provider practice preferences, which are likely to be important predictors of the number of people in contact with mental health services. There is some consistency across England in how services are provided compared to other countries, as in all areas the provider is the NHS, but there will be differences in the population coverage of services and clinician supply that was not possible to investigate or take account of in this study.

Data for this study was obtained from Public health England fingertips data which likely represents the best available routinely collected data on this area. However other studies have highlighted the generally poor quality of NHS routine data in mental health services, an issue highlighted in recent work examining the rising number of detentions under the mental health act [43]. For the majority of variables data was not available at the same time point as the outcome (see Table 1) and so the most recent data was used, therefore the validity of this data will be affected by to what degree these variables have changed over time. The prevalence data for severe mental illness was likely of good quality, as it was based on GP register data, but the prevalence of common mental disorders was model based. The model uses national survey estimates from the Adult Psychiatric Morbidity Survey (APMS) applied to local population demography (age, sex and deprivation) [6]. The estimates have been shown to be consistent with other estimates of CMD prevalence such as those produced by NHS Digital. However the calculations will not capture all of the true variation in CMD prevalence and Public Health England acknowledge that the estimate is likely an under-estimate, as it is based on individuals living in private households, excluding those who are homeless or in institutional settings [6]. The data available on ethnicity at CCG level had clear limitations, as all non-White ethnicities were categorised together, making associations with ethnicity different to interpret. Ethnicity at CCG level was last measured in 2011 during the census and so this has likely changed to a degree since then. Future data collection should include measures disaggregated by different ethnicities.

The size of the unit of analysis was large and previous work has suggested that ward level, which are much smaller than CCGs, is too large to determine the effects of ‘place’ on individual risk of mental disorders [7]. However the unit of analysis used is relevant when considering how local commissioning and service structure may impact use of services. No data was available on the level of substance use within CCGs which could be potentially related to unemployment, prevalence of mental disorders and demand for mental health services. Substance use has previously been found to be an area level predictor for the number of admissions to psychiatric hospital at the district level in England [44].

### Conclusions and implications

There is substantial variation in the use of mental health services by area of England, which is not well-explained by previous research. Social factors including deprivation, unemployment and the ethnic makeup of the population continued to be associated with the number in contact with mental health services after controlling for the prevalence of mental illness. This suggests that there may be factors that influence the local population use of mental health services independent of the prevalence of mental disorder. More deprived areas with higher unemployment will likely need service provision above what might be estimated based on the burden of disease alone. The study also suggests that greater access to primary care psychological therapy in an area is not related to a reduction in secondary mental health service use, although scaling up these services can be justified on other grounds. Additionally, this study has identified a possible inequality in access to services based on ethnicity, which warrants further investigation.

## Supplementary Information

**Additional file 1: Appendix 1**. Missing data. **Appendix 2** Results of Moran I test for spatial autocorrelation.

## Data Availability

The datasets analysed during the current study are available at Public Health England Public Health Profiles https://fingertips.phe.org.uk/, Office of National Statistics Population Estimates https://www.ons.gov.uk/peoplepopulationandcommunity/populationandmigration/populationestimates/adhocs/009301/populationestimatesmedianages/foradministrative/electoraland/censusgeographies and Office for National Statistics Standard Area Measurements https://geoportal.statistics.gov.uk/datasets/standard-area-measurements-2019-for-health-areas-in-england

## Abbreviations

APMS: Adult Psychiatric Morbidity Survey
BAME: Black and minority ethnic
CCGs: Clinical Commissioning Groups
CMD: Common mental disorder
CQC: Care Quality Commission
IAPT: Improving Access to Psychological Therapies
IMD: Index of Multiple Deprivation
NHS: National Health Service
ONS: Office for National Statistics
PHE: Public Health England
SMI: Severe Mental Illness
