# Supplementary material for: Investigating geographical variation in the use of mental health services by area of England: a cross-sectional ecological study
Source: BMC Health Serv Res. 2021 Sep 11;21:951. doi: 10.1186/s12913-021-06976-2 (PMC8434730; doi:10.1186/s12913-021-06976-2)
Supplement: Supplementary file 1 — Additional file 1: Appendix 1. Missing data. Appendix 2 Results of Moran I test for spatial autocorrelation. [file 12913_2021_6976_MOESM1_ESM.docx]

APPENDIX- ‘Investigating geographical variation in the use of mental health services by area of England: a cross-sectional ecological study’

Authors:

1. Dr Lucy Maconick, Division of Psychiatry, University College London
2. Dr Luke Sheridan Rains, Division of Psychiatry, University College London
3. Rebecca Jones, Division of Psychiatry, University College London
4. Dr Brynmor Lloyd-Evans, Division of Psychiatry, University College London
5. Professor Sonia Johnson, Division of Psychiatry, University College London *

*Corresponding author. Corresponding author address: Professor Sonia Johnson, Division of Psychiatry, University College London, 6^th^ Floor Maple House, 149 Tottenham Court Road W1T 7NF

Appendix 1: Missing data

|  | Number (%) of CCGs with data |
| --- | --- |
| Primary outcome and population | 194 (100%) |
| Age | 194 (100%) |
| Gender | 194 (100%) |
| %BAME | 194 (100%) |
| imd | 186 (96%) |
| unemployment | 194 (100%) |
| popdensity | 188 (97%) |
| mhqof | 193 (99%) |
| Estimated prev cmd | 194 (100%) |
| Access to iapt | 192 (99%) |
| Iapt recovery | 194 (100%) |

Appendix 2: Results of Moran I test for spatial autocorrelation

| Variables | I | E(I) | Sd(I) | z | p-value |
| --- | --- | --- | --- | --- | --- |
| Residuals model 1 | -0.003 | -0.006 | 0.012 | 0.177 | 0.430 |
| Residuals model 2 | -0.003 | -0.006 | 0.012 | 0.175 | 0.431 |
| Residuals model 3 | -0.000 | -0.006 | 0.012 | 0.444 | 0.328 |
| Residuals model 4 | -0.001 | -0.006 | 0.012 | 0.395 | 0.347 |
| Residuals model 5 | 0.000 | -0.006 | 0.012 | 0.468 | 0.320 |

*Appendix 2: Results of Moran’s I test for spatial autocorrelation applied to the residuals of the models. Model 1: Multilevel model for association of population density with number of people in contact with mental health services, adjusted for age, prevalence of common and severe mental disorders. Model 2: Adjusted model for ethnicity Model 3: Adjusted model for IMD Model 4: Adjusted model for Unemployment Model 5: Full model*
